# Entropy-Based Multifractal Testing of Heart Rate Variability during Cognitive-Autonomic Interplay

**DOI:** 10.3390/e25091364

**Published:** 2023-09-21

**Authors:** Laurent M. Arsac

**Affiliations:** Univ. Bordeaux, CNRS, Laboratoire IMS, UMR 5218 Talence, France; laurent.arsac@u-bordeaux.fr

**Keywords:** cardiovascular, multifractality, DFA, autonomic control, heart–brain, central autonomic network, cognitive task

## Abstract

Entropy-based and fractal-based metrics derived from heart rate variability (HRV) have enriched the way cardiovascular dynamics can be described in terms of complexity. The most commonly used multifractal testing, a method using q moments to explore a range of fractal scaling in small-sized and large-sized fluctuations, is based on detrended fluctuation analysis, which examines the power–law relationship of standard deviation with the timescale in the measured signal. A more direct testing of a multifractal structure exists based on the Shannon entropy of bin (signal subparts) proportion. This work aims to reanalyze HRV during cognitive tasks to obtain new markers of HRV complexity provided by entropy-based multifractal spectra using the method proposed by Chhabra and Jensen in 1989. Inter-beat interval durations (RR) time series were obtained in 28 students comparatively in baseline (viewing a video) and during three cognitive tasks: Stroop color and word task, stop-signal, and go/no-go. The new HRV estimators were extracted from the f/α singularity spectrum of the RR magnitude increment series, established from q-weighted stable (log–log linear) power laws, namely: (i) the whole spectrum width (MF) calculated as α_max_ − α_min_; the specific width representing large-sized fluctuations (MF_large_) calculated as α_0_ − α*_q+_*; and small-sized fluctuations (MF_small_) calculated as α_q−_ − α_0_. As the main results, cardiovascular dynamics during Stroop had a specific MF signature while MF_large_ was rather specific to go/no-go. The way these new HRV markers could represent different aspects of a complete picture of the cognitive–autonomic interplay is discussed, based on previously used entropy- and fractal-based markers, and the introduction of distribution entropy (DistEn), as a marker recently associated specifically with complexity in the cardiovascular control.

## 1. Introduction

Over the years, complexity metrics derived from cardiovascular signals, e.g., heart rate variability (HRV) or blood pressure fluctuations, have enriched how the neurophysiological control of cardiovascular dynamics can be described. Perhaps the most striking observation after decades of methodological development lies in the fact that there exists no universal method and probably no unique kind of complexity in cardiovascular dynamics. Cardiovascular control involves oscillatory components of vagal and sympathetic modulations that combine to form complex dynamics. Cognitive tasking generates interconnections between cortical/subcortical networks and cardiovascular centers in the brainstem, reflected in HRV dynamics, as described by markers of so-called complexity [1,2]. In this domain, metrics obtained from entropy- or fractal-based analyses of HRV have demonstrated obvious interest in informing beyond the autonomic behavior per se on subtle adaptations in brain–heart interplay.

Entropy-based complexity is believed to grasp the information content in the HRV signal by quantifying the degree of predictability or regularity [3]. Somewhat differently, by adopting the point of view of fractal calculus, scaling properties in HRV quantify the way fluctuation sizes depend on observational timescales [4]. To characterize this dependence, analytic methods have envisaged windowing the entire series of collected data into subparts called “bins”, and evaluating a given statistical property in these bins (subparts) against the same property in other, larger bins. The most used method, detrended fluctuation analysis (DFA), regresses the variance in bin samples against a variety of bin sizes on a log–log scale to obtain a scaling exponent α-DFA, defined as the slope of this linear relationship [5]. An extension of DFA consists in determining whether α-DFA (the slope) keeps the same value when modifying the fluctuation function using q moments, which emphasizes large-sized (q > 0) or small-sized (q < 0) fluctuations in the analyzed series [6]. The heterogeneity in q-dependent α-DFA indicates a multifractal rather than a monofractal behavior of the measured series. It has often been stressed that biological signals exhibit multifractal behaviors [7], which deserved methodological efforts for a quantitative approach of multifractality.

To obtain a multifractal geometry, an alternative method to DFA exists that introduces the concept of entropy [8]. In brief, rather than bin variance, the method has its root in bin proportion (P), where P_i_(L) is the amount of the measure, i.e., the sum of the samples in the i-th bin of size L, divided by the amount of all the measure across the entire series, i.e., the sum of all samples in the series (see Appendix A for an example). Then, the collected series is deformed using a predetermined set of q exponents to emphasize large-sized (q > 0) and small-sized (q < 0) fluctuations. The multifractal spectrum is defined thanks to pairs of f(q,L) and α(q,L), obtained after a mass coefficient (µ_i_) is calculated for each scale and each q moment as:

(1) $${µ}_{i}\left(q,L\right)=\frac{{\left[P_{i}\left(L\right)\right]}^{q}}{\sum_{i}^{}{\left[P_{i}\left(L\right)\right]}^{q}}$$

The singularity strength α(q) is the singularity for µ(q)-weighted P(L), estimated by

(2) $$\alpha \left(q\right)=\underset{L\to 0}{\lim}\frac{\sum_{i}^{}{µ}_{i}\left(q,L\right){logP}_{i}\left(L\right)}{\log L}$$

The estimates α(q) belong to the multifractal spectrum if the Shannon entropy of μ(q,L) evolves with L along a dimension f(q) calculated as

(3) $$f\left(q\right)=\underset{L\to 0}{\lim}\frac{\sum_{i}^{}\;{µ}_{i}\left(q,L\right)log{µ}_{i}\left(q,L\right)\;}{\log L}$$

Generally, a set of predetermined values of q is used (see Section 2.3), including extreme q values that provide P-L relationships not compatible with a linear fit. Based on a threshold linear correlation coefficient as a benchmark, only the values of q providing stable (linear) scaling relationships served to obtain the multifractal spectrum [9]. The spectrum is determined by the curve α,f and the main estimator provided via the method is the spectrum width (MF), calculated as MF = α_max_ − α_min_, thus representing the range of singularities α present in the measured signal.

The method has been successively applied in psychology to explore cognition through movement behavior. Establishing the link between multiplicative cascading and nonlinear multifractal behavior in this complex movement system helped conceptualize executive control as an emerging, nonlinear structure, where the activity of the system components can be assembled and disassembled by the interactions unfolding across multiple scales [8,10]. A behavior of similar nature was shown in postural control [11,12]. To date, the nature of the emerging structure in cardiovascular control during brain–heart interactions has not been described in similar terms, and what cardiovascular complexity really means in this context is not a settled matter. In a recent study that specifically addresses this notion, distribution entropy (DistEn) in HRV has been suggested as a reliable estimate of complexity in a human model of spinal cord injury [13]. It is hoped that entropy-based multifractal testing can complement the rather fuzzy picture of cardiovascular complexity and that applications to cognitive tasks can help highlight specific aspects of HRV complexity. For that, the present reanalysis of the HRV series collected during Stroop, stop-signal, and go/no-go tasks could help complete a picture in which multiscale sample entropy and multiscale multifractal estimators showed specific links with cognitive interference (Stroop), action cancellation (stop-signal), and movement restraint (go/no-go) [2]. Perhaps a multifractal testing combining entropy concepts/calculations may help to capture subtle differences in HRV during cognitive tasks.

Here, it is hypothesized that multifractal testing of HRV using the method of Chhabra and Jensen may provide reliable estimators to complete the picture of cardiovascular complexity. To validate this hypothesis, multifractal testing based on Shannon entropy was used for a reanalysis of HRV measures obtained during cognitive tasks. Some links between task and specific HRV metrics have already been shown by applying multiscale sample entropy and multiscale–multifractal DFA to the same dataset so that new information is expected here.

## 2. Materials and Methods

### 2.1. Subjects and Data Collection

Among 37 participants in the study of Bouny et al. [2], we considered only the 28 participants having RR series > 513 samples to finally obtain an RR-increment series with a length of 512 samples. The experimental protocol, which is described in detail in [2], consisted in recording RR series from a bipolar electrode transmitter belt Polar H10 (Polar, Finland) while participants watched an emotionally neutral video (baseline), then performed successively in a randomized order, a Stroop color-word task (Stroop), a go/no-go task (go/no-go), and a stop-signal task (stop signal), all displayed on a computer screen. Briefly, the Stroop task consisted in hitting a keyboard key as fast as possible that corresponded to the ink color of a word naming another color. The test assesses the ability to inhibit cognitive interference. Go/no-go assess the ability to restrain an action thanks to the relative amounts of no-go signals, here 30%. For this, a green or a red ellipsis appeared on the screen indicating ‘press the space bar’ (go) or ‘do not press the space bar’ respectively. Reaction time in the ‘go’ condition is presented as critical. The stop-signal task consisted in displaying an arrow pointing to the left or to the right to trigger a fast response by hitting the left or right key on the keyboard. The stop signal was presented in a short unpredictable delay (50 to 400 ms) after the presentation of the go stimulus, so that the response of the participant is already in the process of completion. So, the task assesses the ability to cancel an action as it imposes the suppression of an already initiated motor response. Each task lasted approximately 8 min with a one-minute pause between each. The participants kept a sitting position during the whole experiment (±40 min). As already attested in the initial publication, the study was approved by the IRB of the faculty des STAPS and followed the rules of the Declaration of Helsinki.

### 2.2. Measured Series, Analyses, and Main Parameters

The RR series that were free of visually removed artifacts and having length > 513 samples were truncated from the center of the series, which corresponded to the middle of the task. Differentiated series were obtained from HRV recordings and transformed to contain non-negative values by taking the absolute value of the successive increments:

$$\left|∆{RR}_{i}\right|=abs({RR}_{i+1}-{RR}_{i})$$

Differentiating provides signals resembling multiplicative cascades, and the absolute value avoids negative numbers that could not contribute to the computation of bin proportion and for which the logarithm is not defined [6,9].

A predetermined list of bin length (L) was set up for the need of the analysis, which corresponded to the observational time scales: 4, 8, 16, 32, 64, and 128 samples. So, the minimal observational scale contained four samples, and the maximal scale contained N/4 samples.

A predetermined set of q moments was considered with q values ranging from −20 to 20. The range of q values was established from a first-round analysis showing that a wider range was unnecessary due to the obvious lack of (log–log) linearity in the corresponding relationships between bin proportion probability and the scales in all the series (see below).

### 2.3. Main Steps in the Employed Method

For readers who would like to go through the method of Chhabra and Jensen [14] in detail, a tutorial is available in a must-read paper published recently [9]. In short, here, for each bin length L (4 to 128) and each q moment (−20 to 20), the sum of the $\left|∆{RR}_{i}\right|$ samples in the bin i was divided by the sum of the samples of the whole series to obtain the bin proportion for each i segment, $P_{i}\left(L\right)$ in Equations (1) and (2). Then, the sum of the ${\left[P_{i}\left(L\right)\right]}^{q}$ was divided by the sum of the ${\left[P_{i}\left(L\right)\right]}^{q}$ of the whole series (Appendix A).

To obtain a direct estimation of the multifractal spectrum, the Chhabra and Jensen method considers three critical characteristics of bin proportion: (i) the singularity strength α quantifies the scaling between bin proportion P and bin length L by establishing the linear relationship logP~logL; (ii) using q moments to weight bin proportions allows quantifying heterogeneity in scaling exponents, thus providing a q-dependent range of α values; (iii) in addition, this heterogeneity is also sensitive to bin length. It follows that the (negative) Shannon entropy of bin proportions regressed against bin length quantifies this sensitivity (scaling exponent) in terms of the Hausdorff dimension, f. The method of Chhabra and Jensen is described as a direct determination of the multifractal spectrum, where DFA-based methods need to use a Legendre transformation [15,16].

As introduced in Section 1, the q-order generalization of the singularity strength α is the slope of the µ-weighted P against L on a log–log scale. In the same vein, the q-order generalization of the Hausdorff dimension f is the slope of the Shannon entropy (actually the negative Shannon entropy) against logL.

By considering the paired value of α and f at the given q value, the multifractal spectrum is obtained by plotting f(q)~α(q). For that first step, a range of q values [−20 +20] was used. However, it is advised to hold only q values that allow obtaining relative stability in the µ(q)logP~logP and µ(q)logµ(q)~logP linear relationships to establish the final multifractal spectrum. It is achieved by considering a benchmark, here a given value of R^2^ as a threshold below which linearity cannot be an acceptable fitting model to quantify α and f. Although the most recommended threshold R^2^ > 0.9 was used here, it is generally advised to choose the threshold value as a function of the analyzed series and the parametric data. Other standards exist to determine the boundaries of the multifractal spectrum that have been employed, for instance, with DFA-based methods of multifractal testing [17,18,19,20]. Again, all these considerations are nicely discussed in the recent tutorial, including the procedure for surrogate data testing [9].

### 2.4. Phase-Randomized Synthetic Data

The width of the multifractal spectrum (here MF) has its possible origin in non-linearly decomposable forms of tightly interwoven interactions involving processes across all scales on the one hand. On the other hand, multifractality can be observed in a case of interactions unfolding similarly but across several independent scales (see Figure 1 in [8]). It is possible to distinguish between across-scale origins, and independent-scale origins of multifractality by transforming the original series into phase-randomized surrogates that retain only the linear structure of the series. This is generally achieved using the iterative amplitude-adjusted Fourier-transform (IAAFT) routine proposed by Schreiber and Schmitz [21]. The MF of a finite set of IAAFT series (here, 50 surrogates obtained after 100 iterations to preserve the spectral amplitude despite phase randomization) is compared to the MF of the original series using a one-sample *t*-test [9].

### 2.5. DistEn

**Figure 1:**
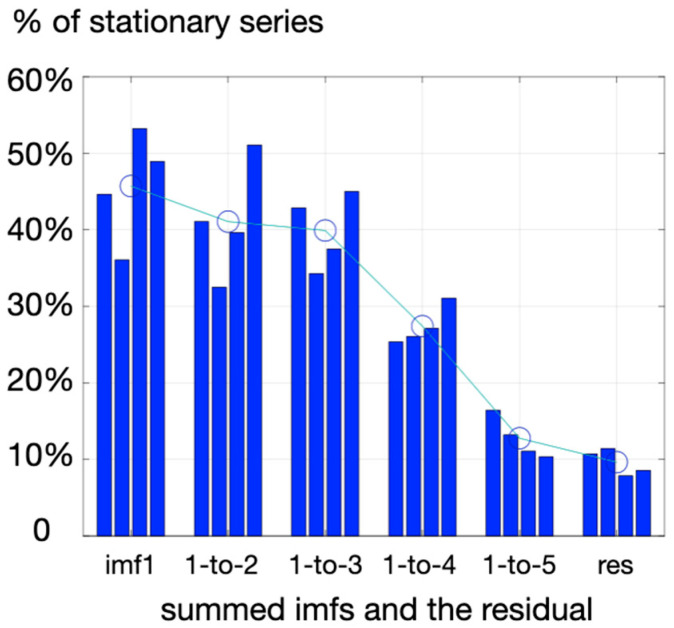
Percentage of stationary series identified via the RWS method when summing successive imfs (e.g. 1-to-2, then 1-to-3, then 1-to-4) obtained from prior HRV mode decomposition via the emd procedure. Imf1 contains the highest frequencies; imf4 adds low frequencies that could resemble trends identified as poor stationarity, given the short signal. The four bars indicate the four conditions, respectively: rest, Stroop, stop-signal, and go/no-go. Wide circles indicate the averaged % of stationary signals when grouping the four conditions.

A recent publication shows an interest in computing DistEn to infer complexity in HRV series [13]. The workflow allowing the computation of this estimator is explained in the cited work, and the code used here was kindly provided by the authors. Computations of DistEn were obtained here to analyze the complexity of the HRV series obtained at rest and during each cognitive task. Keeping in mind that any computation of entropy is affected by trends or drifts in the measured signal, a filtering pre-processing step is mandatory, and it should be added that the process is critical when it comes to the use of entropy markers [13,22]. Especially when analyzing nervous autonomic regulations of the heart rhythm, so-called very low frequencies (VLF < 0.04 Hz), despite obvious physiological backgrounds, are out of the spectrum of interest when it comes to capturing the entropy linked to short-term sympathovagal modulations. For this, a data-driven filtering method was used by employing empirical mode decomposition [23]. The method extracts the dominant oscillations in the measured signal based on local maxima to provide so-called intrinsic mode functions (imf) and a residual that does not have the property of an imf (meaning that it does not exhibit symmetric oscillations around zero, as did intrinsic modes, imfs). As it is generally unclear if subtracting only the residual is enough to detrend the signal adequately, the criterion here to provide a stable (not drifting) final signal to explore DistEn was a test of stationarity, and the RWS method as proposed by Porta et al. [24]. By progressively adding imf1 (highest frequency), with other imfs of lower frequencies (imf2, imf3, …) and testing at each step how many experimental series reached reasonable stationary, a threshold was observed when summing three imfs (imf1-3) rather than four imfs (imf1-4) (Figure 1). So, DistEn was computed here on a series of pre-processed HRV retaining the first three imfs obtained with the emd procedure.

The present computation of DistEn considered all existing two-sample segments and evaluated all the corresponding distances between two points of the series (embedding dimension m = 1). Then, to derive the Shannon entropy of all the distances that are computable in the HRV series, the empirical probability distribution function (ePDF) of the distances was calculated over 512 bins to derive the Shannon entropy of the distances, ShEn. DistEn was obtained by normalizing ShEn by log2(512), where 512 is the number of bins considered.

### 2.6. Statistics

Given the design of the experiment, the aim was to evaluate HRV estimators obtained in repeated cognitive tasks of different natures performed by all the participants, which means a four-repetition design in one sample of individuals. So, after testing for normality using the Shapiro–Wilk test to determine if the null hypothesis of composite normality is a reasonable assumption regarding the population distribution (based on *p*-value < 0.05), either a one-way ANOVA for repeated measures (null hypothesis true) or a Friedman test was used to compare the estimators among the four tasks.

## 3. Results

### 3.1. MF Estimates

**Figure 2:**
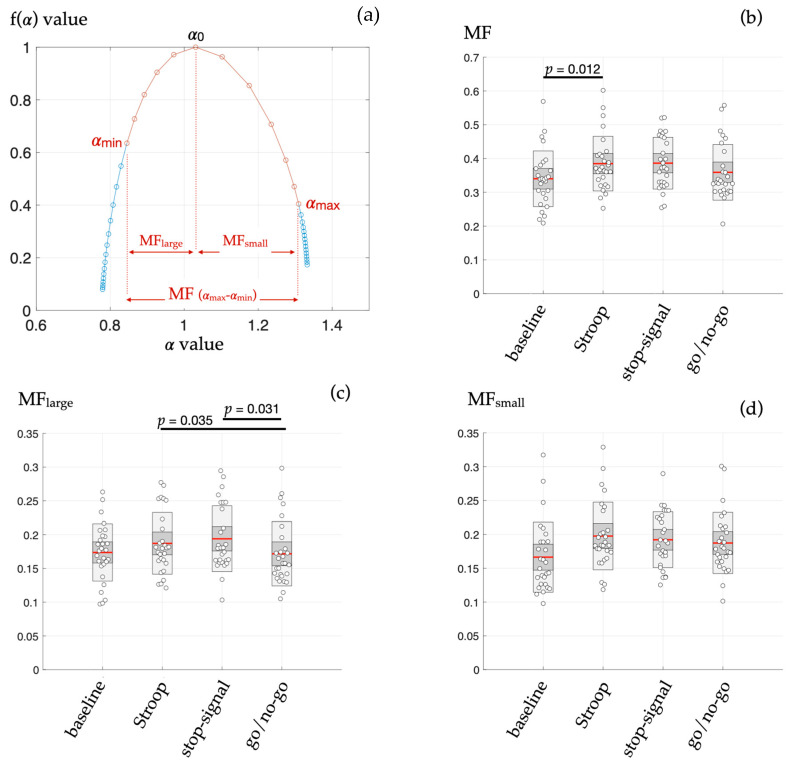
(**a**) A typical multifractal spectrum obtained by using a full range of q values [−20:20] (blue dots) where only q values providing R^2^ > 0.9 (see methods) were retained (red dots) for computing the value of estimates MF, MF_small_, and MF_large_. (**b**–**d**) Values obtained from the three main estimates of the HRV behavior during each condition, baseline, and three cognitive tasks: MF, the multifractal spectrum width; MF_large_, the width of the spectrum that is determined by magnifying large-sized fluctuations in the signal when using q > 0 moments.; MF_small_, the width corresponding to q < 0 moments and magnification of small-sized fluctuations. Points are layed over a 1.96 SEM (95% confidence interval) in heavy grey and a 1 SD in light grey. The mean is indicated as a red line; *p*-values are indicated when the difference between the experimental conditions is significant in post-hoc tests.

Figure 2 shows a typical MF spectrum and compares the multifractal estimates for the measured RR increment series during each cognitive task. As main results, the figure highlights a different multifractal cardiovascular control behavior among cognitive tasks and resting conditions reflected in MF (*χ*^2^ = 12.66, *p* = 0.005, post-hoc indicated on the figure) and in MF_large_ (*χ*^2^ = 10.91, *p* = 0.012, post-hoc indicated on the figure). While cognitive inhibition required to perform the Stroop test was specifically reflected in the MF estimator, MF_large_ allowed distinguishing the go/no-go task from the other tasks. A higher MF_large_ during go/no-go indicated that large-sized fluctuations in HRV are specifically affected by the kind of cognitive–autonomic interplay in this particular condition. It has been suggested that the spectrum symmetry could be another estimator of interest [25]. The MF_large_/MF_small_ ratio (*sym*) was computed to assess this symmetry. The value obtained for *sym* showed no difference (*χ*^2^ = 2.63, *p* = 0.452) among the four conditions (rest 1.09 ± 0.32, Stroop 0.98 ± 0.28, stop-signal 1.03 ± 0.23, and go/no-go 0.94 ± 0.23).

### 3.2. Surrogate Data Testing

Phase-randomization of increment RR series, a method that aims to distinguish across-scales vs. scale-dependent origin of multifractality, indicated that across-scale behavior was not really dominant. In fact, the difference between the MF in measured series and MF in their linearized surrogates (IAAFT) did not reach significance, respectively, *p* = 0.061, *p* = 0.091, *p* = 0.076, and *p* = 0.069 for Stroop, stop-signal, and go/no-go. The corresponding t-statistic index was not higher either. In the same way, MF_small_ and MF_large_ values were not modified via the phase-randomization procedure (all *p* > 0.05).

### 3.3. DistEn

DistEn in the detrended HRV series showed almost identical values at rest (0.883 ± 0.015) and during each task: Stroop (0.872 ± 0.020), stop-signal (0.874 ± 0.020), and go/no-go (0.874 ± 0.019). Hence, despite a slightly higher value in resting condition, DistEn failed to distinguish task-dependent HRV complexity.

## 4. Discussion

Multifractal testing has been the subject of intense research in recent years to describe the complexity of control systems, including posture [12], cognition [10,26,27], movement [19,20,28], and cardiovascular control [17,18]. The present study, elaborating on a dataset obtained during cognitive tasking, aimed at exploring multifractal estimates of HRV complexity derived from a direct determination of the multifractal spectrum based on Shannon entropy metrics: the Chhabra and Jensen method [14]. Exploring new estimates is based on the intuition that different aspects of complexity can be reflected by specific markers, which depends on how entropy-based or fractal-based metrics are computed, as illustrated in recent studies [2,13]. Specifically, different components of the multifractal spectrum reflecting large-sized and small-sized fluctuations in HRV were evaluated here and confronted with cardiovascular responses to four different cognitive challenges that rely on distinctive cognitive–autonomic interplay (Figure 2). Cognitive models are obvious candidates to explore cardiovascular complexity since brain–heart interplay is generally more prompt to alter the complex dynamics of HRV rather than to produce a simple shift in the sympatho–vagal balance reflected in the power spectral density of HRV [1,29,30,31].

The main findings here, thanks to the application of the Chhabra and Jensen method to directly obtain the multifractal spectrum of HRV, were that: (i) during the Stroop task only, the width of the multifractal spectrum (MF) was greater than in baseline (resting) conditions (Figure 2b); (ii) the subpart of the MF spectrum highlighting the large-sized fluctuations in HRV (MF-large) mainly distinguished specific HRV dynamics during the go/no-go task (Figure 2c). It is worth noting that no such methodological sensitivity was reached in a previous analysis of this dataset [2] based on refined composite multiscale (sample) entropy, linear multiscale entropy (LMSE), conditional entropy computed with the binning model-free estimator (CEBi), conditional entropy computed with the kernel model-free estimator (CEKe), conditional entropy computed with the nearest neighbor model-free estimator (CENN), or the multifractal–multiscale detrended fluctuation analysis (MM-DFA). In fact, RCMSE and MM-DFA showed promising results, indicating that system complexity rather than signal randomness alone is modified, but estimates derived from said methods unveil singular HRV dynamics in one cognitive task each, separately. The ability of the present multifractal testing to provide estimates that distinguish several situations could be plaid for better performance, at least for the exploration of heart–brain interactions.

Applying the method of Chhabra and Jensen on increments series allowed a non-ambiguous determination of multifractal spectra, as illustrated in Figure 2. The predetermined choice of range in *q* moments (−20 to +20) allowed obtaining coherent values of *q*-dependent singularity strength α (*x*-axis) as well as coherent values of fα (*y*-axis) based on Shannon entropy conceptualization. The use of a correlation coefficient benchmark ensuring that stable values of α and fα are used to draw the multifractal spectrum allowed providing estimators MF, MF_large_, and MF_small_ adequately. In this vein, combining better performance and non-ambiguous application, the present method is suggested to offer a reliable additional (but not exclusive) tool for exploring cardiovascular complexity.

Recently, distribution entropy was compared to sample entropy and fuzzy entropy in HRV dynamics, following the intuition that specific estimates of HRV might be less blind to complexity structures or more adequate to highlight a certain form of complexity [13]. Interestingly, the authors compared DistEn in white noise, pink noise, brown noise, and chaos to experimental HRV series from a human model to explore several forms of complexity. It was shown that DistEn highlights complexity in a chaotic series as well as in HRV series obtained in low-level spinal cord-injured (SCI) participants [32]. In our conditions, DistEn failed to provide a distinction among the cognitive tasks; the resting period before tasking also showed slightly but not significantly higher DistEn values (see Section 3.2). It is concluded that MF and DistEn may not provide the same type of information; although DistEn is an obvious marker of HRV complexity emerging from perturbated dynamics in vascular districts of SCI patients, this estimator might be less sensitive to changes in HRV complexity during cognitive tasks. In agreement with this intuition, sample entropy (RCMSE) distinguished the cognitive tasks in the previous analyses of the present data, and Castiglioni et al. [13] opposed sample entropy and DistEn. So, as hypothesized during the introduction of the present work, the notion that HRV markers derived from different nonlinear analyses can point to subtle neurophysiological dynamics in cardiovascular control coordination is strengthened by the present findings. Discrepancies among nonlinear analyses are not alarming but rather encourage us to consider that the dynamics of biological systems may take different forms of complexity.

The hypothesis that different kinds of emerging interactions in biological systems can be reflected in subtle multifractal properties is attractive. Nonlinear interactions are suspected to cohere in the large system linking cognitive, sensory, and motor processes, which has been documented in several studies, some of them being cited in the present manuscript. Less attention has been paid to subtilities in nonlinear dynamics of cardiovascular control. Mainly, it is unknown whether multifractality in HRV reflects intricate interactions across a wide range of scales or if scale-specific non-overlapped multifractal phenomena are finally reflected in a spectrum of similar width. Here, surrogate data testing using phase randomization to preserve only the linear attributes of the series leaves MF, MF_small_, and MF_large_ unchanged. This would indicate that the strength of nonlinear multifractality in our conditions is low, so the multifractal organization mostly emerges from an assemblage of local interactivity spanning several scales [9]. In a sense, this echoes the need to combine a multifractal and a multiscale approach to HRV dynamics [17]. This observation offers another interesting point of view when considering why MF but not DistEn could distinguish cognitive tasks. The MF in our condition could be sensitive to changes in local interactivity during mental tasking, while DistEn might rather point to nonlinear behavior of the cardiovascular control when peripheral vascular beds have ineffective autonomic control. It could be added that MF in the present dataset analysis resembles RCMSE (sample entropy) in the previous exploration of the same dataset [2] since both estimates distinguish the HRV signature of cognitive interference during the Stroop test. On its side, MF-large, a specific aspect of MF where large-sized fluctuations are emphasized, is distinguished mainly from the go/no-go situation from other cognitive tasks. Large-sized fluctuations in a RR increments series rely dominantly on intense vagal outflow because only vagal arousals can have a rapid action on the sinus rhythm. Establishing the link between action–restraint (go/no-go testing) and multifractality in intense vagal outflow is out of the scope of the present study, but it is the perfect illustration that adding a multifractal approach based on Shannon entropy could shed light on particular aspects of cardiovascular control, not encountered in other approaches. Among alternative methods, multifractal DFA has been a widely used and reliable method to explore cardiovascular complexity. It is worth noting that DFA-based approaches evaluate the power-law form of the power spectrum, while the present approach evaluates the power-law form of an aggregate distribution of fluctuations. The former indicates the magnitude of oscillations at different observational scales, and the latter reflects the probability of different-sized fluctuations. Both approaches can make significant contributions to exploring cardiovascular complexity since the present reanalysis of the dataset illustrates that different kinds of mathematical views of the HRV series can help gain an improved understanding of complex neurophysiological mechanisms.

## 5. Conclusions

The observations in this work are consistent with the initial idea that there is no unique estimate(s) that can help researchers grasp the HRV complexity, but rather a number of mathematical views of the HRV series, each able to provide a reliable estimator of self-organized interactions in cooperating systems. This work is further evidence that entropy-based, multifractal-based, and other methods are not mutually exclusive but provide complementary information to understand the functioning of a complex networked system. In the absence of direct evidence to link a given marker to a given organization of cardiovascular control, our only weapon is default reasoning, which is strengthened by multiplying complexity explorations in specific contexts. MF, MF_small_, and MF_large_ derived from applying the Chhabra and Jensen method to HRV could add value to this ongoing way of research.

## Data Availability

Data can be supplied by the author on reasonable request.

## Appendix A

A more detailed explanation about calculating bin (signal subparts) proportions (P) at a given bin length (L) is provided here. Indeed P~L and q-P~L relationships are the roots that allow the evaluation of α and f(α). A range of observational timescales is chosen, where each timescale corresponds to a subpart (here called bin) of the whole series containing a corresponding number of samples. For each timescale, the sum of the samples in the bin is divided by the sum of the samples of the whole series to obtain the bin proportion (P). An example is provided assuming that bins contain four samples (L = 4) each (RR increments in ms) and supposing that the whole series has eight samples (for simplicity). Actually, longer collected series allow us to reiterate the same calculation at several bin lengths (observational scales) chosen to be equally distant on a log scale (e.g., L = 8, L = 16, L = 32, … L = N/4).

The whole series = 48 4 13 14 6 28 2 12

bin1 = 48 4 13 14      bin2 = 6 28 2 12

sumbin1 = 79        sumbin2 = 48

sumseries = 79 + 48 = 127

P1 = 79/127 = 0.622     P2 = 48/127 = 0.378

The q moments are then introduced to modify each initial bin proportion and obtain new q-specific P; an example is provided assuming q = −4.

P1^−4 = 6.681        P2^−4 = 48.982

q-sum = 55.663

q-P1 = 6.681/55.663 = 0.120  q-P2 = 48.982/55.663 = 0.880

The above example (q = −4) illustrates that q < 0 values enhance small-sized fluctuations: here, P2 was only 0.378, but q-P2 reached 0.880. Inversely, P1 was 0.622, but q-P1 dropped to 0.12. The inverse is true; when using positive q moments (example here with q = 3) one obtains a magnification of large-sized fluctuations:

P1^3 = 0.241         P2^3 = 0.054

q-sum = 0.295

q-P1 = 0.241/0.295 = 0.817   q-P2 = 0.054/0.295 = 0.183
